# Cortico-Muscular Coherence Is Reduced Acutely Post-stroke and Increases Bilaterally During Motor Recovery: A Pilot Study

**DOI:** 10.3389/fneur.2019.00126

**Published:** 2019-02-20

**Authors:** Richard Krauth, Johanna Schwertner, Susanne Vogt, Sabine Lindquist, Michael Sailer, Almut Sickert, Juliane Lamprecht, Serafeim Perdikis, Tiffany Corbet, José del R. Millán, Hermann Hinrichs, Hans-Jochen Heinze, Catherine M. Sweeney-Reed

**Affiliations:** ^1^Neurocybernetics and Rehabilitation, Department of Neurology and Stereotactic Neurosurgery, Otto-von-Guericke University, Magdeburg, Germany; ^2^Department of Neurology, Otto-von-Guericke University, Magdeburg, Germany; ^3^MZEB, Pfeiffersche Stiftungen, Magdeburg, Germany; ^4^MEDIAN Klinik, Neurological Rehabilitation Center, Magdeburg, Germany; ^5^Institute for Neurorehabilitation, Affiliated Institute of the Otto-von-Guericke University, Magdeburg, Germany; ^6^Defitech Chair in Brain-Machine Interface, Ecole Polytechnique Fédérale de Lausanne, Geneva, Switzerland; ^7^Brain-Computer Interfaces and Neural Engineering Laboratory, School of Computer Science and Electronic Engineering, University of Essex, Colchester, United Kingdom; ^8^Department of Behavioral Neurology, Leibniz Institute for Neurobiology, Magdeburg, Germany; ^9^German Centre for Neurodegenerative Diseases, Magdeburg, Germany

**Keywords:** cortico-muscular coherence, EEG, EMG, stroke, rehabilitation, wrist

## Abstract

Motor recovery following stroke is believed to necessitate alteration in functional connectivity between cortex and muscle. Cortico-muscular coherence has been proposed as a potential biomarker for post-stroke motor deficits, enabling a quantification of recovery, as well as potentially indicating the regions of cortex involved in recovery of function. We recorded simultaneous EEG and EMG during wrist extension from healthy participants and patients following ischaemic stroke, evaluating function at three time points post-stroke. EEG–EMG coherence increased over time, as wrist mobility recovered clinically, and by the final evaluation, coherence was higher in the patient group than in the healthy controls. Moreover, the cortical distribution differed between the groups, with coherence involving larger and more bilaterally scattered areas of cortex in the patients than in the healthy participants. The findings suggest that EEG–EMG coherence has the potential to serve as a biomarker for motor recovery and to provide information about the cortical regions that should be targeted in rehabilitation therapies based on real-time EEG.

## Introduction

Stroke is one of the leading causes of disability, ranking third in the world as a contributor to disability-adjusted life years (DALYs) (1). Over the 20 years from 1990 to 2010, stroke incidence and absolute numbers of stroke survivors have increased dramatically, with the number of survivors in the age group over 75 years showing a drastic rise (2). Better understanding of the mechanisms involved in motor recovery has the potential to lead to improved approaches to rehabilitation. Evidence suggests that the functional recovery achieved after stroke involves different cortical areas taking over the function of those that are damaged, including ipsilateral motor cortex. For instance, increased cerebral blood flow in sensorimotor cortex and both parietal lobes has been observed on positron emission tomography (3, 4). Functional magnetic resonance imaging has also revealed greater activation in patients post-stroke both in the same motor regions as healthy participants and also in the ipsilateral hemisphere (5). Enhanced understanding of how different brain areas are involved in motor recovery after stroke should lead to improved targeting of these regions in rehabilitation programmes.

Motor deficits are widely thought to result from a loss of functional connectivity between motor cortex and musculature. The aim of rehabilitation is to restore this function, either by re-establishing this connectivity or supporting the development of alternative brain–muscle connectivity. Cortico-muscular coherence (CMC) between brain electrical activity (electroencephalogram: EEG) and electrical activity recorded from muscle (electromyogram: EMG) has been proposed as a potential biomarker reflecting the regain of muscular control by cortex (6). Brain-computer interfaces (BCI) and transcranial electrical stimulation (TES) are receiving growing attention as rehabilitation approaches in which motor recovery is promoted by neurofeedback based on recordings of or direct manipulation of brain electrical activity (7–14). These therapies are based on an ability to identify cortical regions engaged in movement noninvasively and to monitor their activity associated with movement or movement attempts in real time.

A robust association has been observed between oscillatory rhythms in the motor cortex and movement (15–19). EEG–EMG coherence provides a measure of functional connectivity and could reflect the extent to which a particular motor cortical area is able to generate limb movement. Firstly, coherence was observed between local field potentials recorded from motor cortex and EMG signals in macaques that was modulated by movement (20). Second, EEG–EMG coherence has been found to increase with motor learning in healthy individuals (21, 22). Third, a reduction in EEG–EMG coherence has been observed following stroke (6, 23), and a recent case study suggested that quantifying EEG–EMG coherence could provide a measure of recovery of motor function post-stroke (24). EEG–EMG coherence thus represents a potential biomarker for monitoring regain of function during rehabilitation. Here we expand these findings to a larger patient group, evaluating CMC over the course of motor recovery and also comparing CMC at the final evaluation with that in healthy participants. We present here a preliminary study investigating the clinical progression and parallel CMC changes through the subacute and chronic phases in a group of 4 patients who had suffered an acute, left-hemispheric ischaemic stroke and compare their recovery with CMC in 7 healthy volunteers. We also present a case of right-hemispheric ischaemic stroke. On the basis of the current findings, we provide an estimate of the number of patients that would need to be recruited for a study in which stroke rehabilitation over the first months post-stroke is compared between treatment groups.

## Methods

### Participants and Design

We recorded simultaneous EEG and EMG data from 7 right-handed healthy volunteers, and from 4 left-sided stroke patients (see Table 1 for handedness) and one right-sided, right-handed stroke patient during the acute and subacute recovery phase following stroke. All participants were seated in front of a screen, with their forearms comfortably rested on a table in front of them, and visual cues were presented via Presentation (Neurobehavioral Systems, USA). Both groups received the instruction to extend their wrist when an arrow pointed upwards and to remain still and relaxed when the arrow pointed downwards. The healthy participants and the right-sided stroke patient were instructed to perform continuous, self-paced movements during movement trials. Each movement trial was followed by a rest trial. 16 rest condition trials and 16 movement condition trials of the right hand were recorded, with one trial lasting 20 s. The left-sided stroke patients were asked to extend or attempt to extend the right wrist once on presentation of an arrow pointing upwards and to rest when the arrow pointed downwards. The arrows were accompanied by a bar moving upwards for movement and downwards for rest. For the patients the up arrow was green and the down arrow was red, but otherwise, for both groups, the visual stimuli for movement and rest were identical except for the direction of the arrow. The movement of the bar downwards during rest was to ensure that visual cortical activation did not differ between movement and rest conditions. Right hand movement and movement attempts were evaluated for all participants except for the right-sided stroke patient, in whom left hand movement and movement attempts were also studied. This study was carried out in accordance with the recommendations of the Local Ethics Committee of the Otto-von-Guericke University, Magdeburg. Approval was granted for data collection and analysis from patients and from healthy participants for separate studies evaluating motor function using EEG and EMG. All patients and healthy participants gave written informed consent in accordance with the Declaration of Helsinki. The stroke patients were enrolled in a multi-center BCI-based rehabilitation study, and the healthy participants were enrolled as a part of a separate, preceding study, intended to inform the BCI study. The patients presented here are a subset of the patients recruited so far at the University Clinic, Magdeburg, and include all those with a left-sided subcortical stroke. The initial plan was to employ repeated movements in the BCI study, to maximize the classification rate of movement attempts in the early phase of rehabilitation, in which movement may be minimal or absent, on the basis of successful classification of imagined movement with this approach (25, 26). One patient (right-sided stroke patient: male, 56 years old, ischaemic, subcortical stroke to right centrum ovale, right-handed with Edinburgh Handedness Inventory Score of 68) performed this paradigm during piloting of the rehabilitation study. The decision was subsequently taken to employ single movement attempts, because this method is more natural (27), and thus potentially offers a more physiological approach to re-establishing functional connectivity between cortex and muscle. The paradigm was then altered for the subsequent patients, including the 4 left-sided subcortical stroke patients presented here. In order to provide continuous visual feedback to the patient regarding the success of the movement attempt, a moving bar was chosen. The downward movement was used for rest, so that visual stimulation would be similar for both conditions. A color difference was deemed unlikely to yield detectable EEG differences and was used to make it easier for patients to know the current condition. We additionally present CMC during movements of the affected and the unaffected hand the pilot data from the single patient as these data provide a bridge between the paradigms employed.

**Table 1:** Clinical data from patients.

**Patient**	**EHI**	**Lesion location**	**Session 1**		**Session 2**		**Session 3**	
			**Time post-stroke**	**FMA: wrist, FMA/66**	**Time post-stroke**	**FMA: wrist, FMA/66**	**Time post-stroke**	**FMA: wrist, FMA/66**
1	−20	Subcortical: left medial internal capsule, and re-infarction directly behind, in dorsolateral internal capsule.	8 days	0, 10	7 weeks, 1 day	5, 38	11 months	10, 65
2	0	Subcortical: left Internal capsule.	6 days	0, 8	7 weeks	0, 11	12 months	10, 58
3	100	Subcortical: small lacunar infarct in posterior part of left internal capsule.	13 days	0, 16	7 weeks, 2 days	9, 61	6.5 months	10, 62
4	−78	Cortical: left central motor area. Cortical, occipital left. Unclear whether new or old. Subcortical: old, bilateral small lacunar infarction in white matter.	20 days	0, 20	6 weeks, 4 days	2, 47	15 months	7, 53

*EHI, Edinburgh Handedness Inventory. Mean age 58.3 years (range 52–64 years)*.

**Figure 1:**
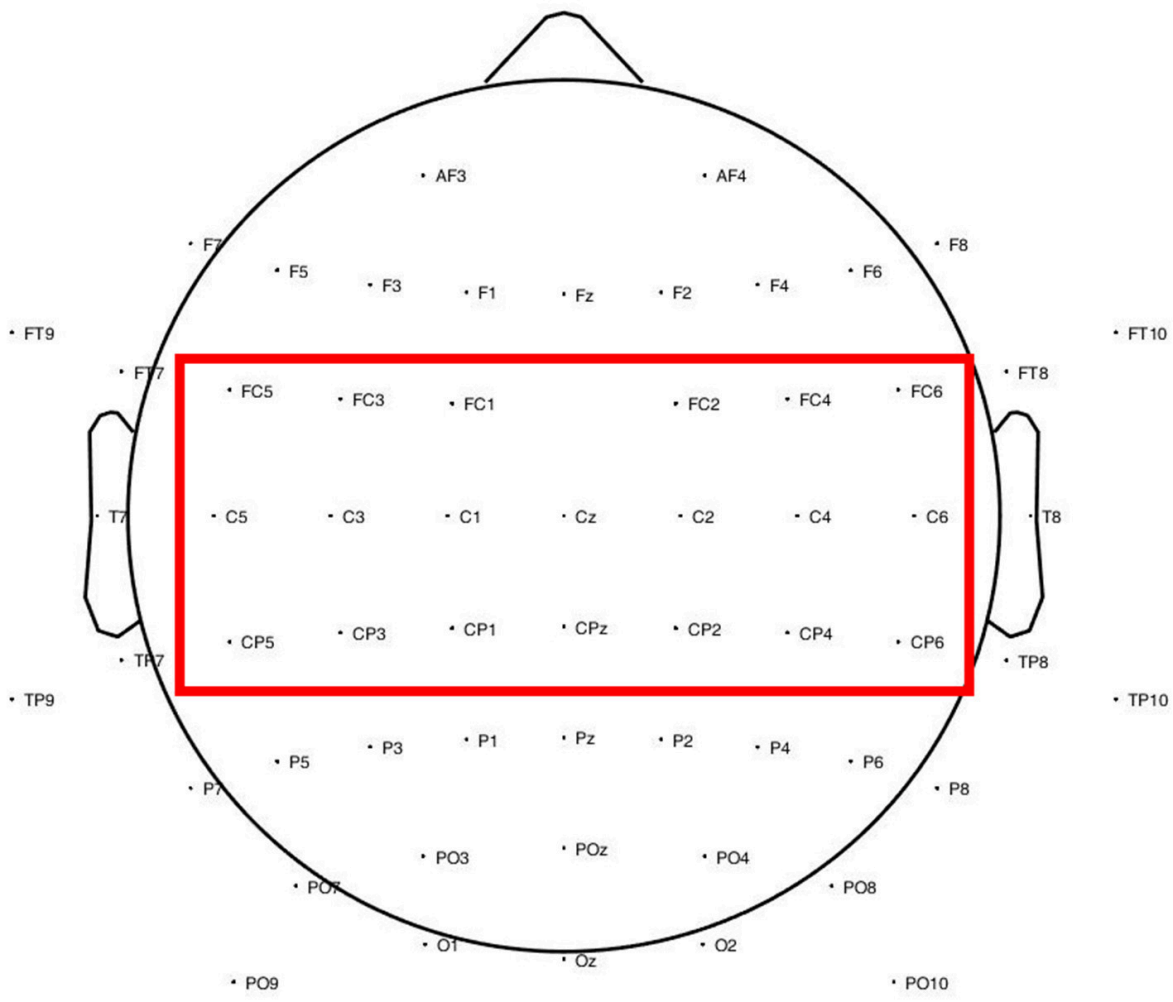
Electrode locations according to the 10-10 system. The electrode locations over motor cortex are indicated by the red box.

Scalp EEG recordings were made using an EEG cap from Brain Products. EEG electrodes were placed according to the 10-10 system montage (Figure 1). Data were recorded against an FCz reference. 4 of the 64 electrodes (FP1, FP2, AF7, AF8) were used for bipolar EMG recording from wrist extensor muscles (2 electrodes for each hand). Electrodes were placed approximately 5 cm apart over the wrist extensor musculature as determined by palpation on wrist extension. Data were digitized with a sampling rate of 500 Hz.

Impedance in the healthy participant group in the data analyzed (motor cortex electrodes: FC5, FC3, FC1, FC2, FC4, FC6, C5, C3, C1, Cz, C2, C4, C6, CP5, CP3, CP1, CPz, CP2, CP4, CP6) was below 5 kΩ. In the patient group, the impedance levels were higher due to caution taken to avoid excessive skin abrasion in hospital in-patients. This approach was considered to be of additional importance in an EEG-based BCI study, in which daily electrode application was required for the therapy sessions and was deemed essential to minimize the study dropout rate, which is known to be high in acute stroke patients. The impedance in the patient group was mainly kept below 10 kΩ and did not exceed 50 kΩ (except at FC1 in Patient 1, Session 1: 77 kΩ–no significant CMC was identified at this site in Session 1 but also not in Session 2, when the impedance at FC1 was 1 kΩ). A recent event-related potential (ERP) study comparing ERPs from high- with low-impedance recordings, reported a reduced signal-to-noise ratio (SNR) when recording commenced with an impedance >30 kΩ (28). In the current study, impedance exceeded 30 kΩ for Patient 1 at 5 electrode sites (FC1, FC2, FC6, C5, CP5) in Session 1 and at no electrode sites in Session 2, for Patient 2 at 2 electrode sites (CP3, CP5) in Session 1 and at 2 electrode sites in Session 2 (C2, CP6), for Patient 3 at no electrode sites in either Session, and for Patient 4, at 3 electrode sites (C1, CPz, CP6) in Session 1 and at 3 electrode sites (FC2, C1, CP2) in Session 2. When the patients returned to the clinic for Session 3, the impedances did not exceed 20 kΩ. Impedance did not exceed 30 kΩ at any electrode site for the right-sided stroke patient. Given the amplifier’s input impedance of 10 MΩ, it should be noted that impedance does not result in a reduction in recorded signal amplitude (29, 30), and a lower SNR in ERP calculation simply resulted in a requirement for more trials to yield significant findings (28). We do not expect our examination of the temporal relationship between EEG and EMG signals to be affected by impedance, given that signals were successfully recorded.

The patient inclusion criteria were an infarction diagnosed on structural magnetic resonance imaging (MRI) lasting more than 24 h, resulting in a score of 0–2/5 on the Medical Research Council power scale for wrist movement. Exclusion criteria were aphasia or cognitive impairment to an extent that prevented ability to understand and perform the task, medication with L-dopa, and severe pain, fatigue, or depression. All patients received individualized rehabilitation therapy in the Neurorehabilitation Centre, Magdeburg. Patients underwent a clinical assessment and EEG–EMG recordings at Session 1: several days after stroke (11.75 ± 5.40 days), Session 2: 7 weeks ± 1.87 days after stroke, and a longer-term follow-up at Session 3: 11.3 ± 3.04 months post-stroke. The main outcome measure was the motor function section of the upper extremity evaluation of the Fugl-Meyer Assessment (FMA-UE) (24) with a maximum score of 66 points. We also evaluated the wrist section of the FMA separately. It involves 5 items, where movement was scored from 0 to 2, 0 being no movement and 2 being full movement, resulting in a maximum of 10 points. FMA scores were compared across sessions using the Friedman test.

### Pre-processing

For all analyses, Matlab, version 2015b (The Math Works, USA) was used. The data recordings were imported using EEGLAB (31). First, data epochs were extracted during right hand movement and rest. Using the Current Source Density toolbox (32), a Laplacian Filter was applied to each dataset to improve spatial resolution of the signals. The Laplacian filter was constructed using a current source density transformation (32) The radially flowing current, passing from the cortex beneath the electrode into the skull and scalp, was estimated for each electrode location, using a spatially weighted sum of the potential gradients directed at this location from all other electrode sites. The scalp Laplacian used was derived from the negative second spatial derivative of the interpolated scalp surface potentials, resulting in reference-free, spatially-enhanced potentials. This step was followed by applying a 1–200 Hz bandpass filter and a 49–51 Hz notch filter to eliminate line noise. EMG recordings were re-referenced to provide a bipolar recording for each side. As McClelland et al. showed that rectification was an unnecessary step in EMG pre-processing (33), unrectified EMG data were used for our analysis.

Data recorded from healthy participants during 16 movement epochs and 16 randomly selected rest epochs were concatenated, respectively, to provide a time series for each condition. Recordings were taken from second 3 to 18.36 (15.36 s), in order to include only data during which there was actual movement during all the movement episodes, resulting in one time series comprising 16 times 15.36 s (= 245.76 s) for each condition. These data were then windowed into 2.048 s epochs. Data containing artifacts, determined by visual inspection and using EEGLAB‘s automated artifact rejection with default settings (threshold voltage: 1,000 μV, probability threshold: 5 standard deviations), were excluded from subsequent analysis. The artifact inspection was performed by RK and JS, under the supervision of CS, who is experienced in EEG artifact inspection. Blinding to data sets was not deemed necessary, because CMC could not be estimated in raw EEG and EMG data. Independent component analysis (ICA) was performed to identify and remove eye-blink artifact components. Rejection criteria for ICA components were unipolar frontal representations on component topographic channel plots (topoplots), additionally to identification of eye blink-related activations on visual inspection. After artifact rejection, movement data were aligned in 2.048 s windows according to movement onsets. The Teager-Kaiser energy operator (TKEO) (34, 35) was calculated according to the following formula:

(1) $$\begin{array}{r} TKEO\left(n\right)=\;x^{2}\left(n\right)-x\left(n+1\right)x\left(n-1\right) \end{array}$$

for the movement data and rest data of the corresponding EMG channel, depending on the hand that was moved. *x* is the EMG voltage in the frame number *n*. If the energy operator in movement data exceeded 10 standard deviations of the energy operator of the rest data for at least 100 consecutive bins (0.2 s), a movement onset was defined. Data from −512 to 512 bins surrounding onsets were extracted, resulting in 2.048 s time windows.

As movement for the left-hemispheric stroke patients was not self-paced but cued, their recordings were not aligned to movement onsets but to cue signals. 2 s windows, starting from 1 s after cue presentation, were used for analysis. Artifact removal was conducted in the same manner as described for the healthy participant data.

### Coherence

For coherence analysis, the movement epochs were concatenated to a single time series. Coherence spectra for every combination of scalp electrode with EMG electrode of the moved hand were calculated using the multitaper method implemented in the Neurospec toolbox for Matlab (Neurospec, Version 2.0, 2008, see (36) for a theoretical framework). Non-overlapping windows consisting of 1,024 frames (2.048 s) were used to estimate and average frequency domain auto-spectra of the EEG and EMG signals in the time series. The spectra were calculated at a frequency resolution of 0.49 Hz per bin. Coherence was calculated as shown in Equation 1, expressed as the magnitude squared correlation coefficient between two signals:

(2) $$\begin{array}{r} |R_{x\;EMG}^{2}|=\frac{|S_{x\;EMG}\left(f\right){|}^{2}}{S_{xx}\left(f\right)S_{EMG\;EMG}\left(f\right)} \end{array}$$

where |*S*_*xEMG*_*(f)*|, the estimated cross-spectrum between scalp electrode *x* and the EMG electrode at frequency *f*, is analogous to the covariance. *S*_*xx*_*(f)* and *S*_*EMGEMG*_*(f)*, the estimated auto-spectra at frequency *f*, are analogous to the variance of each signal (36). However, artifact rejection resulted in different numbers of trials for healthy participants and patients, which can affect the strength of coherence. In order to eliminate the effect of trial length on coherence, we performed bootstrapping in a similar fashion to Carlowitz-Ghori et al. (37), using 5,000 repetitions. After determining the smallest number of trials available (*n* = 41), coherence spectra were assessed as the mean of 5,000 bootstrap spectra calculated from *n* randomly selected trials for each patient and healthy participant. One Hundred permutations were performed, calculating coherence for stacked 2 s sequences with randomly selected starting points. Coherence involving a particular EEG electrode at a frequency was deemed significant if the bootstrapping estimate exceeded 95% of the values of the permutations in that channel and frequency.

In the following calculations, significant coherence values in the beta band (12–30 Hz) over motor cortex electrodes (FC5, FC3, FC1, FC2, FC4, FC6, C5, C3, C1, Cz, C2, C4, C6, CP5, CP3, CP1, CPz, CP2, CP4, CP6), as shown in Figure 1, were included. The number of significant bins, reflecting the spatial extent and the frequency range involved in CMC, was determined by counting the number of EEG electrode sites over the motor cortex at which coherence was deemed significant from bootstrapping and permutation in the steps described above, for each of the 37 frequency bins in the beta band. We examined the level of coherence: peak coherence values over the motor electrodes during right hand movement were extracted for further statistical analysis. We also investigated the ratio of coherence between the affected and unaffected hemispheres of the patients and compared the findings to those of the healthy participants.

Laterality indices were calculated according to Equation 3:

(3) $$\begin{array}{r} LI=\frac{Q_{Left\;hemisphere}-Q_{Right\;hemisphere}}{Q_{Left\;hemisphere}+Q_{Right\;hemisphere}} \end{array}$$

where *Q* is the maximum coherence in the corresponding hemisphere. Laterality indices could range from −1 to 1, with positive values indicating a lateralization to the left (lesioned) hemisphere and vice versa. Friedman Tests for repeated measures were conducted to compare patient data across three Sessions. Significant results from Friedman tests were further analyzed with *post-hoc* Wilcoxon rank sum tests.

In order to compare session 3 patient data to data from healthy participants a one-sided ANOVA was performed with the factor *Group* (healthy participant/stroke patient), following Levene’s tests where parameters were first tested for significant differences in variance between patient and healthy participant data. If there was no significant difference in variance (*p* > 0.05), an ANOVA was performed.

Correlation was calculated between clinical outcome measure (FMA-UE and FMA wrist) and the peak beta CMC, for each patient at each of the three assessment times.

On the basis of the change in EEG–EMG coherence over the course of the study, a power estimation was performed, in order to indicate the number of patients that would need to be recruited in a rehabilitation study, such as that comparing use of a BCI with a sham treatment group, in order to achieve 80 % power to detect a difference between groups with a significance threshold of *p* = 0.05.

## Results

### Clinical Evaluation

**Figure 2:**
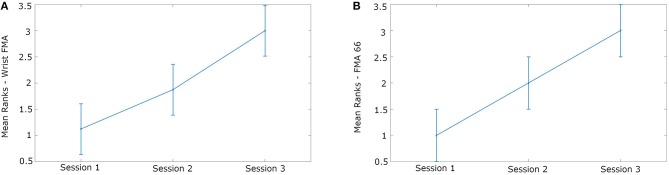
Improvement in upper limb mobility in the patient group, as indexed by **(A)** the wrist-FMA, and **(B)** the motor function section of the FMA-UE score, was observed over the Sessions.

All patients showed an increase in FMA score over the course of the study, with the mean FMA-UE and the mean FMA wrist subsection improving (Table 1). The FMA-UE change over sessions was significant [Friedman’s ANOVA: χ^2^(2) = 8.0, *p* = 0.018], with *post hoc* testing showing a significant increase from 13.5 at Session 1 (directly post-stroke: mean of 11.8 days later) to 59.5 at Session 3 (mean of 11.1 months later) (Wilcoxon test: *p* = 0.029). The mean score in the section of the FMA relating to wrist movement also specifically improved from 0 to 9.75. The improvement in wrist-FMA scores across sessions was significant [Friedman’s ANOVA: χ^2^(2) = 7.6, *p* = 0.022] (Figure 2). *Post-hoc* analysis showed a significant increase from Session 1 to Session 3 (Wilcoxon test: *p* = 0.029), while wrist-FMA did not show significant improvements between Sessions 1 and 2 (*p* = 0.14), and Sessions 2 and 3 (*p* = 0.057).

### CMC

**Figure 3:**
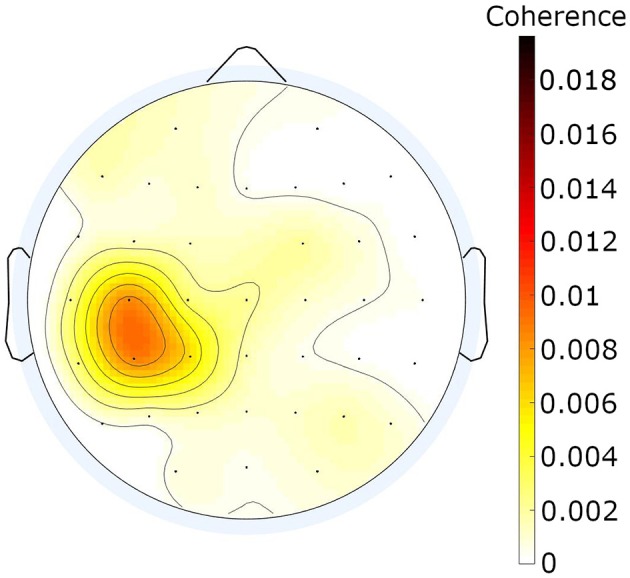
Mean beta EEG–EMG coherence across 7 healthy participants. The values shown resulted from averaging the individual mean coherence patterns in the beta band across all 37 frequency bins across the 7 healthy participants. CMC values determined to be non-significant with the methods described were set to zero. Color scale: CMC.

In the healthy participants, beta EEG–EMG coherence was observed in the contralateral hemisphere during movement (Figure 3). Coherence was deemed significant if the bootstrapping estimate exceeded 95% of the values of the permutations in a specific channel and frequency.

**Figure 4:**
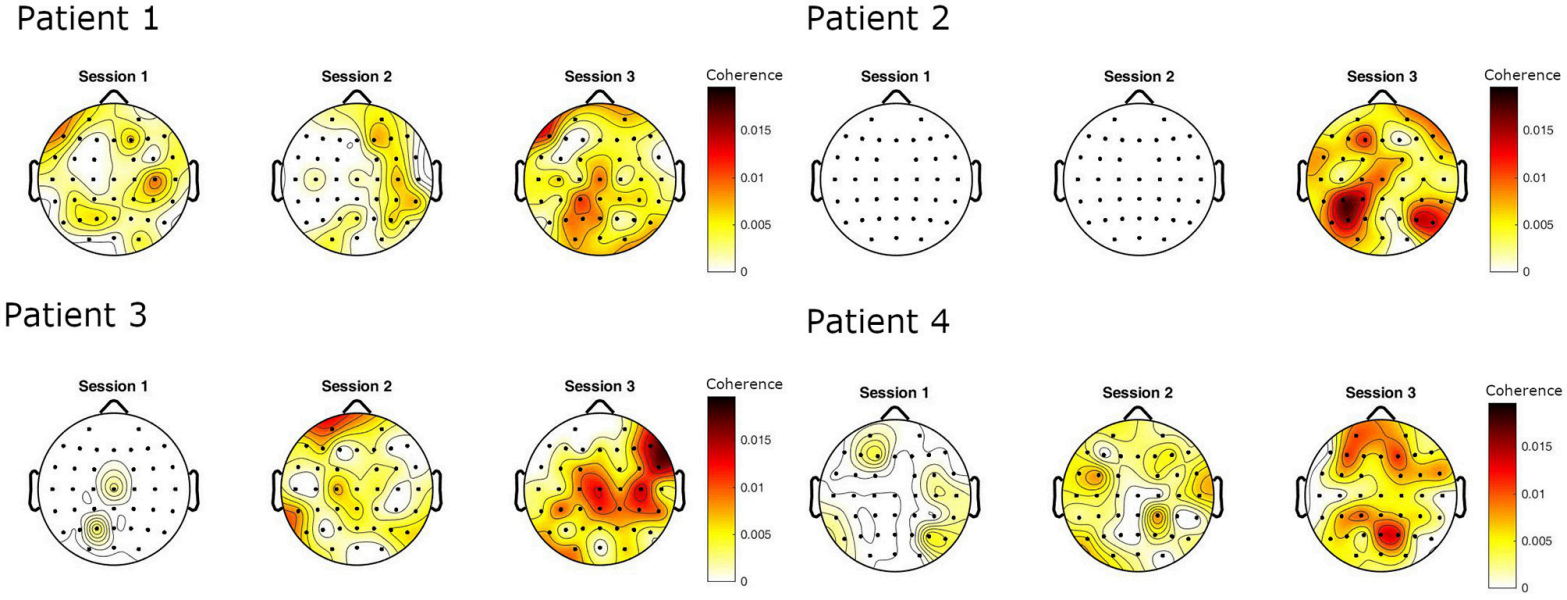
EEG–EMG coherence in the beta frequency range in four patients following stroke, evaluated at three sessions during motor recovery. Color scale: CMC.

**Figure 5:**
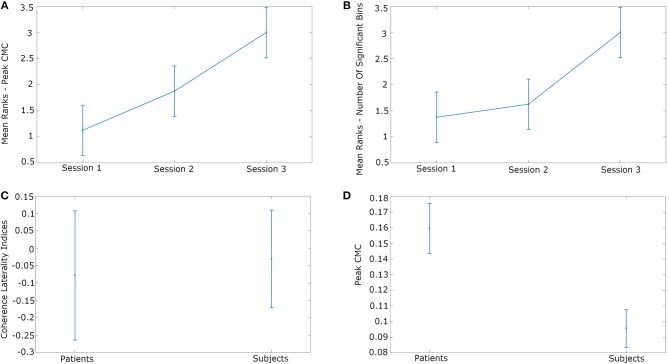
Changes in beta EEG–EMG coherence over time post-stroke. **(A)** Mean ranks of coherence peak in the beta frequency range. **(B)** Mean ranks of number of bins across electrodes and beta frequencies. **(C)** Coherence laterality index (*p* = 0.84). **(D)** Comparison between peak level of beta EEG–EMG coherence between patients on final evaluation and healthy participants (*p* = 0.011).

The EEG–EMG coherence in the beta frequency band in the stroke patients changed over time, both increasing in degree and changing in topographic distribution (Figure 4). The beta peak coherence increased over the course of the three evaluation sessions [Friedman’s ANOVA: χ^2^(2) = 7.6, *p* = 0.022] (Figure 5A). *Post-hoc* Wilcoxon’s rank sum tests revealed that the beta peak coherence in Session 3 was significantly higher than in Session 1 (*p* = 0.029) and Session 2 (*p* = 0.029). The increase from Session 1 to Session 2, however, was not significant (*p* = 0.229). The number of bins across individual beta frequencies (37 frequencies over 12–30 Hz) and motor electrodes (20 electrodes) also increased over the period of observation [Friedman’s ANOVA: χ^2^(2) = 6.53, *p* = 0.038] (Figure 5B). The increase in the number of significant bins in Session 1 and Session 2 was not significant (*p* = 0.49) while the rises from Session 2 to Session 3 (*p* = 0.029) and Session 1 to Session 3 (*p* = 0.029) were significant. The laterality of coherence in patients did not show a significant shift across sessions, however [Friedman’s ANOVA: χ^2^(2) = 0.93, *p* = 0.63]. The degree of laterality of the maximum beta EEG–EMG coherence did not differ between patients at session 3 and healthy participants [one-way ANOVA: *F*(1, 9) = 0.04, *p* = 0.84] (Figure 5C). The peak coherence over motor cortex was higher in patients at the final evaluation session than in the healthy participants [one-way ANOVA: F(1, 9) = 10.22, *p* = 0.011] (Figure 5D).

The mean increase in beta CMC in our patient group was 0.092 (STD: 0.047) from initial evaluation to the third evaluation. On the basis of the current preliminary study, the number of patients that would need to be enrolled in future studies comparing a group participating in a new rehabilitation programme to a control group receiving standard rehabilitation therapy, using a change in peak beta coherence as a marker of improvement, and assuming an improvement equal to the observed standard deviation in the control group, was calculated. To achieve 80% power, with a significance threshold of *p* = 0.05, *N* = 34 patients (17 per group) would be required.

**Figure 6:**
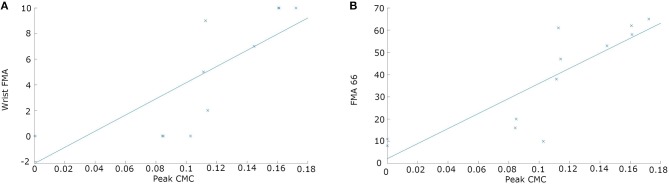
Scatterplots of peak CMC against FMA values **(A)** Correlation between peak CMC and FMA-wrist (*r* = 0.79; *p* = 0.0021). **(B)** Correlation between peak CMC and motor function section of FMA-UE (*r* = 0.84; *p* = 0.0006). Note that each panel contains 12 data points, with three evaluation times for each of four patients. In panel A, two points overlap completely at a CMC peak of 0 with a wrist FMA of 0, and two points are partially overlapping at CMC peaks of 0.085 and 0.016. In panel B, two data points are plotted at a CMC peak of 0.

A correlation was observed between the mean beta peak coherence and FMA-UE (*r* = 0.84; *p* = 0.0006) and also specifically wrist FMA (*r* = 0.79; *p* = 0.0021) (Figure 6). Note that correlation was calculated over values from four patients, each with three evaluation times.

We note that patient 2 had no wrist movement and moreover no detectable movement-related change in the EMG during movement attempts. The plots are thresholded for significant CMC, and no CMC was significant for this patient at these times.

CMC in the alpha (8–12 Hz) and gamma (30–40 Hz) frequency ranges was also increased contralaterally during movement in the healthy participant group but to a lesser extent than the beta CMC (Supplementary Figure 1).

The data recorded during a single recording session from the single right-sided stroke patient (wrist FMA: 4/10; FMA-UE: 32/66) show stronger left-sided and more locally focused beta CMC during right hand movements than right-sided during left hand movements (Supplementary Figure 2).

## Discussion

We identified EEG–EMG coherence in the beta frequency range focused over motor cortex on the contralateral side to hand movement in healthy participants and monitored the development of this marker during the course of motor recovery in patients who had suffered an ischaemic stroke. CMC was initially minimal in the acute recovery phase, then gradually increased over time to reach a greater level than that seen in healthy participants. Moreover, its distribution differed from that found in healthy participants, with CMC observed in both the contralesional (ipsilateral) and the lesioned hemisphere and incorporating both a greater number of frequency bands within the beta range and a larger area of cortex, including posterior cortical areas. These changes in CMC were accompanied by significant motor recovery as evaluated using the wrist subsection of the FMA and the FMA-UE motor function section, with a correlation observed between these FMA scores and the CMC. Our findings suggest that EEG–EMG coherence in the beta frequency range has the potential to be used as a biomarker during stroke recovery. Furthermore, the findings suggest rehabilitation therapies that are steered by EEG activity, such as BCIs and TES, should target broad areas of cortex, with regular updating of the parameters used over the course of the recovery period, as CMC correlated with cortico-spinal communication and was shown to change in its spatial distribution over the course of stroke rehabilitation

Our results are in line with the findings of Rossiter et al. (38). They reported increased CMC in the ipsilateral (contralesional) hemisphere in eight of 25 stroke patients, based on a study examining EEG–EMG coherence in stroke patients. The study involved a large cohort of patients, enabling evaluation of the impact of diverse factors, including subcortical and cortical stroke, as well as different time intervals post-stroke. Some patients had ipsilateral CMC, while others had a peak in CMC in the lesioned (contralateral) hemisphere. These patients did not differ in age, time interval post-stroke, or degree of impairment. Of those with ipsilateral CMC, two had subcortical and six had cortical infarcts. In the current study, we report increasing bilateral CMC in all four of our patients, all of whom had subcortical infarctions and one of whom also had a motor cortical infarction. Evaluation was performed at similar times post-stroke for all patients, and all patients had a similar degree of recovery by the third evaluation. It is likely that the variability in the findings of Rossiter et al. results from their widely ranging cohort, including stroke affecting either hemisphere in both left- and right-handed participants. While handedness was also mixed in our cohort, all patients had a stroke affecting their right side (left brain hemisphere).

Changes in CMC over the course of stroke recovery have also been examined by Carlowitz-Ghori et al. (37). They investigated CMC during movement of the affected and unaffected hands in the acute and chronic stages of stroke in 11 patients. They found a significant decrease in coherence during movement of the affected hand in the acute phase compared to the unaffected hand. In the chronic period, this difference in coherence diminished and peak coherence lessened significantly for the unaffected hand in comparison to the acute period. Contrary to our findings, they did not observe an increase in peak coherence over the time course for the affected hand. However, this could be attributed to the fact that the patients they included had Medical Research Council scores of at least 3, while three of our four patients had no movement at all (reflected by wrist-FMA scores of 0) prior to therapy.

No significant differences in laterality indices were identified in the current study, which is consistent with the findings of a previous study comparing 11 chronic stroke patients (mean years after stroke: 6.5) with nine age-matched participants (39). While Graziadio et al. evaluated absolute laterality, we specifically took account of the side to which lateralization occurred. Contrary to our findings, they did not report significant differences in peak coherence values for healthy participants and patients but rather a dependency between recovery and the degree of symmetry. While our data do not allow evaluation of such a dependency, because all four of our patients achieved similar recovery levels and showed a similar degree of bilaterality of CMC, our findings in this aspect are nonetheless consistent with theirs in terms of CMC showing a bilateral distribution post-stroke.

Our patients showed a more widespread, bilateral activation pattern after motor recovery, with more involvement of the contralesional hemisphere. In contrast, in a study involving chronic stroke patients, only the hemisphere contralateral to the affected hand showed CMC during wrist extension (6). It is plausible that in the more acute phase after stroke, cortical areas neighboring the lesion temporarily take over motor function to compensate for the loss of cortical influence, which may again shift back to a spatial pattern more similar to healthy participants over the course of rehabilitation. We also note that the CMC in our patients was posterior relative to that in the healthy participant group, consistent with findings of increased cortical activity previously reported following stroke (3, 4). A plethora of neuroimmune mediators and biological factors that could index stroke recovery are currently under investigation (40). The engagement of widespread areas of cortex in post-stroke motor recovery may be related to the widespread release of biomolecules following stroke.

The longer time periods after stroke in the aforementioned studies may suggest that the changes we observed are associated with earlier compensatory mechanisms of the central nervous system, while laterality may shift from a high index to the contralesional side in earlier phases of recovery to a more bilateral pattern, as suggested by Bellardinelli et al. (41). They studied eight right-handed patients with stroke affecting the left side. They presented a bi-hemispheric coherence pattern in chronic stroke patients, with increasing amplitude associated with improved clinical performance, as measured with Upper Extremity-FMA after novel BCI-assisted therapy.

The current study has a number of limitations. The direct comparison between data from healthy participants and patients must be interpreted with caution, because the details of the movement paradigms during EEG-EMG recordings differed in the two cohorts compared. While healthy participants performed repetitive, self-paced movements, patients’ movements more closely resembled an isometric contraction due to the difficulty of the task in the early post-stroke period, in which mobility restriction was marked. CMC in the beta band is predominantly described as being associated with submaximal, isometric contractions (37, 38), although it has also been observed in recovering stroke patients involving dynamic muscle contraction (41). The data shown from the single patient who performed the repeated movement paradigm indicate greater contralateral and more lateralized beta CMC during movements of the unaffected wrist than the wrist affected by the stroke. Although coherence in the healthy participant group appeared predominantly in the beta frequency band (alpha and gamma coherence were less marked), the difference in beta peak coherence could also be attributed to the different types of movement instead of solely to cortical plasticity following stroke. The significant CMC–FMA-UE and CMC–FMA-wrist correlations should be interpreted with caution given the low sample number of 4 patients and three measurement points.

Furthermore, the FMA-UE and the wrist section of FMA may not differentiate sensitively enough between the individual levels of rehabilitation in wrist dexterity, as movements are only scored with 0, 1 or 2 points. Although the FMA has proven to be a reliable and efficient assessment of motor function, with high intra- and inter-rater reliability (42, 43), it may not be sensitive enough to the changes in motor performance we are seeking. A ceiling effect for FMA scores regarding the hand has been criticized due to missing out on more complex finger movements (42). Further studies in rehabilitation of motor capacities focusing on rehabilitation of manual dexterity may require a more differentiated scoring system with more discrete levels.

It should also be noted that recordings were performed once in the healthy participant group and three times in the patient group. While we cannot exclude potential alterations in CMC in the healthy participants with repetition of the paradigm, the simplicity of the task makes a practice effect unlikely.

The estimated patient numbers for a future rehabilitation study using peak beta CMC as a biomarker should be considered with caution. The patients involved in this preliminary study received individualized in-patient rehabilitation programmes in the first weeks post-stroke, which likely contributed to their functional improvement and associated CMC. On the other hand, in the absence of an active rehabilitation programme, functional improvement is expected in around half of patients (12).

The current study presents preliminary work, which has the potential to contribute to understanding the mechanisms underlying the plasticity of the link between motor cortex and affected muscles during stroke recovery. Further studies involving greater participant numbers are required to provide plausible answers regarding the role of re-establishing beta-band CMC in the recovery of motor function in the hand.

## Author Contributions

CS-R: study conception and design. RK, JS, and CS-R: clinical and electrophysiological data acquisition. SV and SL: clinical data acquisition. SL, MS, AS, and JL: patient rehabilitation. RK, JS, and CS-R: data analysis and interpretation. SL: data interpretation. RK and CS-R: drafting of manuscript. JS, SV, SL, MS, AS, JL, SP, TC, JdRM, HH, and H-JH: critically revised the manuscript. All authors contributed to planning of patient data collection and read and approved the final manuscript.

### Conflict of Interest Statement

The authors declare that the research was conducted in the absence of any commercial or financial relationships that could be construed as a potential conflict of interest.

## Supplementary Material

Supplementary Figure 1 Topography of CMC during right hand movement in healthy participants. **(A)** CMC in the alpha (8–12 Hz) frequency band. **(B)** CMC in the gamma (30–40 Hz) frequency band were also increased contralaterally during movement in the healthy participant group but to a lesser extent than the beta CMC. Color scale: CMC.

Supplementary Figure 2 Topography of beta-range CMC from the single right-sided stroke patient. The data recorded show stronger left-sided and more locally focused beta CMC. **(A)** Beta CMC was lower over the affected right hemisphere during left hand movements than **(B)** over the left hemisphere during right-handed movements. Color scale: CMC.
