# Supplementary figures and images for: Cortico-Muscular Coherence Is Reduced Acutely Post-stroke and Increases Bilaterally During Motor Recovery: A Pilot Study
Source: Front Neurol. 2019 Feb 20;10:126. doi: 10.3389/fneur.2019.00126 (PMC6391349; doi:10.3389/fneur.2019.00126)

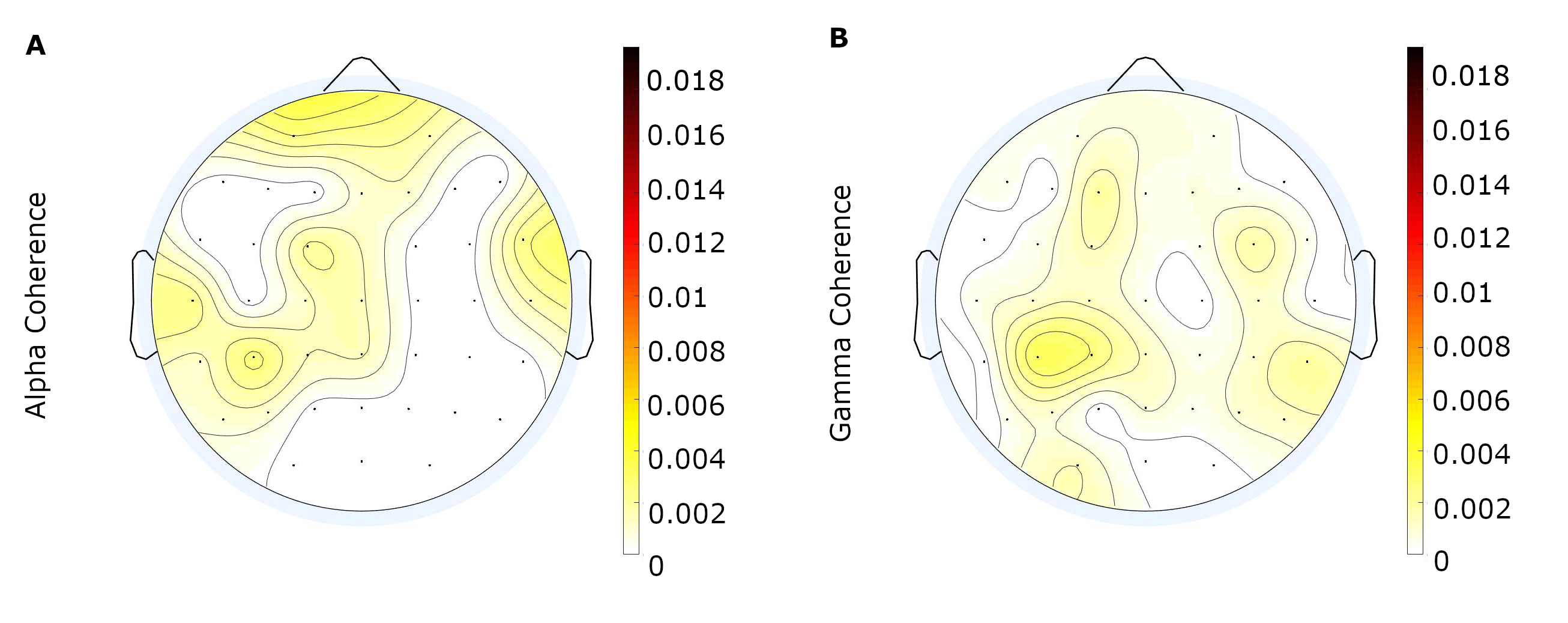

Supplement: Supplementary Figure 1 — Topography of CMC during right hand movement in healthy participants. (A) CMC in the alpha (8–12 Hz) frequency band. (B) CMC in the gamma (30–40 Hz) frequency band were also increased contralaterally during movement in the healthy participant group but to a lesser extent than the beta CMC. Color scale: CMC. [file Image_1.JPEG]

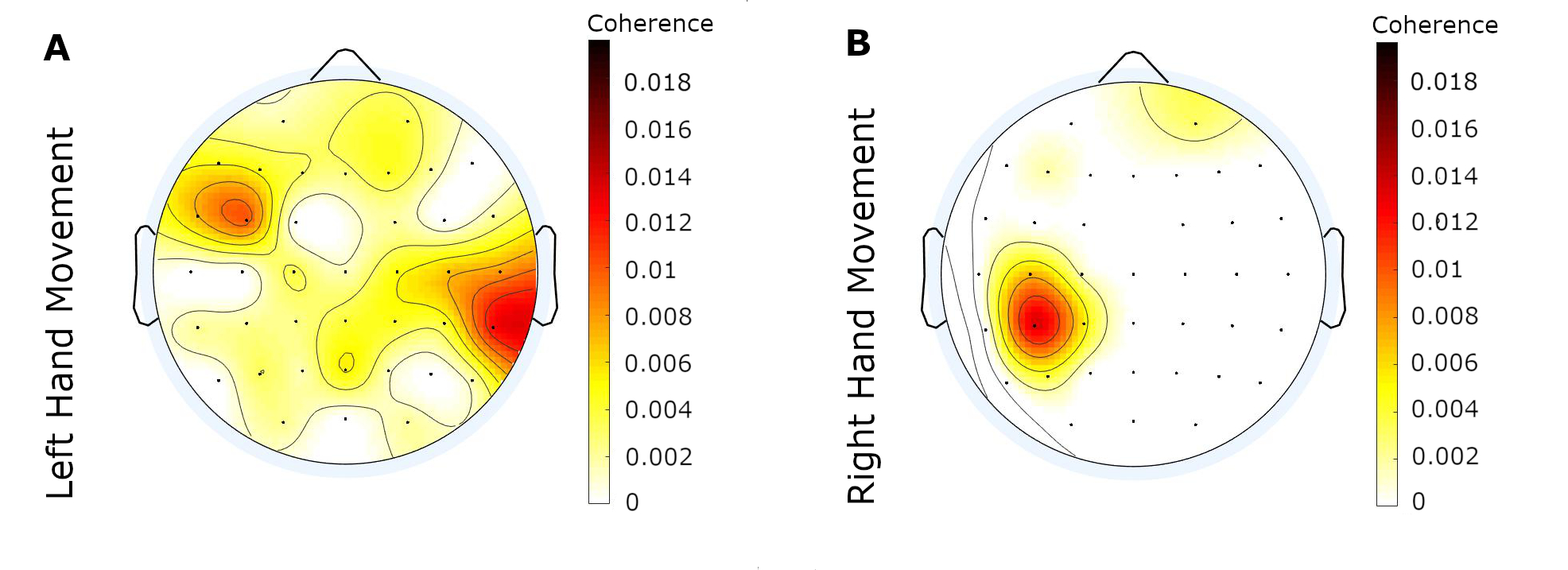

Supplement: Supplementary Figure 2 — Topography of beta-range CMC from the single right-sided stroke patient. The data recorded show stronger left-sided and more locally focused beta CMC. (A) Beta CMC was lower over the affected right hemisphere during left hand movements than (B) over the left hemisphere during right-handed movements. Color scale: CMC. [file Image_2.JPEG]
